# Genome-Wide Identification and Expression Profiling Analysis of the Long-Chain Acyl-CoA Synthetases Reveal Their Potential Roles in Wheat Male Fertility

**DOI:** 10.3390/ijms231911942

**Published:** 2022-10-08

**Authors:** Yongjie Liu, Zihan Liu, Huishu Zhang, Shaohua Yuan, Yanmei Li, Tianbao Zhang, Jianfang Bai, Liping Zhang

**Affiliations:** 1 Institute of Hybrid Wheat, Beijing Academy of Agriculture and Forestry Sciences, Beijing 100097, China; 2 Molecular Genetic Beijing Key Laboratory of Hybrid Wheat, Beijing 100097, China

**Keywords:** long-chain acyl-CoA synthetase, anther, fatty acids, male sterility, cuticular wax

## Abstract

Long-chain acyl-CoA synthetase (LACS), responsible for the conversion of free FAs into acyl-CoAs, is involved in multiple pathways of lipid metabolism. Although LACS genes in Arabidopsis have been well characterized, no detailed information concerning this family is available for wheat. In the present study, a systematic analysis was carried out for the wheat LACS family. As a result, 30 putative *TaLACSs* were identified. Expression analysis revealed that 22 *Takacs* were expressed in wheat anthers. Two orthologs of *AtLACS1*, *TaLACS2* and *TaLACS3*, were repressed at the vacuolated stage in the cold-treated BS366 (a temperature-sensitive genic male-sterile line). Thus, *TaLACS2* and *TaLACS3* may function like *AtLACS1* in wax biosynthesis in anthers, and the repression of both genes may be correlated with the male sterility of BS366. *TaLACS5* is an ortholog of *AtLACS5*, which was expressed exclusively in anthers. *TaLACS5* was repressed in the cold-treated BS366 at the tetrad, uninucleate, and vacuolated stages. The negative correlation between *TaLACS5* and *TaGAMYB*-*B*, and the MYB domain found in the promoter sequence suggested that *TaLACS5* may be negatively regulated by *TaGAMYB*-*B* to participate in wheat fertility. These findings will provide a valuable foundation for the understanding of the wheat LACS gene family in male fertility.

## 1. Introduction

Fatty acids (FAs) are virtually ubiquitous in plant cells, and serve as building blocks for a variety of lipids. FAs are incorporated into membrane glycerolipids, sphingolipids and storage triacylglycerols, where they serve as precursors of surface waxes, cutin and suberin [1]. Previous studies have reported their roles in membrane integrity, energy supply and responses to abiotic and biotic stresses [2,3,4]. Long-chain acyl-CoA synthetase (LACS), responsible for the conversion of free FAs into acyl-CoAs, is involved in multiple pathways of lipid metabolism, such as fatty acid transport, lipid synthesis, and fatty acid β-oxidation [5,6].

Arabidopsis contains one of the largest known LACS families, with nine LACS genes, most of which have been well characterized [1,6]. Characterization of LACS mutants and analysis of expression patterns and subcellular localizations has disclosed a complex network of redundant or moderately redundant LACS activities involved in different aspects of lipid metabolism in Arabidopsis [1]. *AtLACS1*, *AtLACS2* and *AtLACS4* were reported to localize in the endoplasmic reticulum (ER) and participated in the activating of fatty acids for the production of cuticular lipids. *AtLACS1* has a primary role in generating very-long-chain fatty acyl-CoAs that serve as precursors for cuticular wax components. *AtLACS2* seems to function by overlapping with *AtLACS1* in the activation of very long-chain fatty acids (VLCFAs) in the wax component production, and plays a role in the incorporation of C16 and C18 acyl groups into cutin [7,8,9]. *MdLACS2,* from apple, an ortholog of *AtLACS2*, was shown to catalyze the formation of 16:0 CoA [10]. *BnLACS2* from rapeseed involved in seed oil production, exhibited a substrate preference for 14:0, 16:0, 18:0, 18:1, and 22:1 Co [11]. The ER-resident *AtLACS4* was also reported to be partially redundant with *AtLACS1* in providing a substrate for cuticular wax biosynthesis [9]. *AtLACS4* and *AtLACS8* are both ER-localized [12]. *AtLACS9* is the only LACS gene exclusively localized in the outer membrane of the plastid envelope [13]. Both *AtLACS4* and *AtLACS9* are involved in the lipid trafficking between the ER and plastid for glycerolipid synthesis [12]. *AtLACS8* functionally overlaps with *AtLACS4* and *AtLACS9*, as the disruption of *AtLACS8* in the *lacs4lacs9* double mutant results in lethality [12]. In contrast to the above-mentioned LACS members, both *AtLACS6* and *AtLACS7* located in peroxisome have an overlapping role in fatty acid β-oxidation [14,15]. Although no abnormal phenotype was observed for the single mutant of *AtLACS6* or *AtLACS7*, the *lacs6lacs7* double mutant was defective in seed oil mobilization [15]. The function of *AtLACS3* and *AtLACS5* is still unclear. It has been suggested that *AtLACS3* is only expressed in the root, stem, leaf, and flower, whereas *AtLACS5* is exclusively detected in anthers [6,16]. The expression pattern of LACS genes has also been extensively investigated in other plant species. In *B. napus*, 18 of 34 *BnLACSs* expressed in developing seeds [17], which implicated their major roles in lipid metabolism. The expression of *BnLACS4s*, *BnLACS8s*, and *BnLACS9* was quite similar to their closest Arabidopsis orthologs [17]. *MdLACS* in apple are highly expressed in pericarp tissues where wax and cutin are actively produced, which suggests their possible roles in cuticle synthesis [18]. Several *BnLACS5* genes are specifically expressed in buds, anthers, and stamens, which are similar to *AtLACS5* [17]. One cotton ortholog of *AtLACS5*, *GhACS1*, is predominantly accumulated in the anther and plays an essential role in microsporogenesis in the anther development of cotton [19]. Accordingly, it will be intriguing to investigate its role in wheat fertility.

The utilization of heterosis is an important approach to increasing wheat yield, to ensure food security. Known as the prerequisite component of the hybrid system, the male-sterile line directly determines the hybrid yield and seed purity. Thus, a study of the mechanisms underlying male sterility will undoubtedly facilitate its utilization in hybrid breeding [20]. Although the function of LACS and their potential roles in male sterility have been reported in other plants, the role of LACS in wheat still remains unclear. Therefore, a genome-wide analysis of LACS genes will be of great value to understanding the function of the LACS gene family in wheat fertility. In this study, a total of 30 LACS genes were identified through a genome-wide gene family search. Subsequently, the gene phylogenetic relationship, gene structure, protein-conserved domain, chromosome localization, *cis*-acting regulatory elements and expression profiles were systematically analyzed to specify the evolutionary and functional features of *TaLACSs*. In addition, the relative expression of several LACS genes was determined in a temperature-sensitive genic male-sterile (TGMS) line, Beijing Sterility 366 (BS366), to better understand their function in anther and pollen development [21]. This work could contribute to a better understanding of LACS genes in wheat fertility.

## 2. Results

### 2.1. Identification and Annotation of LACS Gene Family Members in Wheat

Nine *AtLACS* protein sequences were used as the query sequence for BLASTP against the wheat genome with an e-value of 1 × 10^−20^. The hidden Markov model (HMM) file for AMP-binding domain (PF00501) was aligned with all the amino acid sequences in the wheat. The obtained AMP-binding genes were filtered, using the Pfam, SMART (http://smart.embl.de/ (accessed on 8 April 2022)), and CDD (https://www.ncbi.nlm.nih.gov/cdd/ (accessed on 10 April 2022)) in NCBI. Eventually, 30 putative LACS family genes were identified from both methods. *TaLACSs* were unevenly distributed on wheat chromosomes, with one–five genes located on each chromosome (Supplementary Table S1), and the number of *TaLACSs* in the A, B, and D genome were the same (10 genes in each sub-genome). The 30 putative *TaLACSs* were renamed *TaLACS1* to *TaLACS30,* based on their chromosomal locations (Supplementary Table S1), and the length of peptides encoded by these *TaLACSs* ranged from 475 amino acids (aa) to 728 amino acids (aa). The theoretical molecular weights of the wheat LACS proteins varied from 52.48 to 79.93 kDa. The theoretical pI values ranged from 5.54 to 8.57 (Supplementary Table S1).

### 2.2. Phylogenetic and Gene Structure Analysis of The LACS Gene Family Members

**Figure 1:**
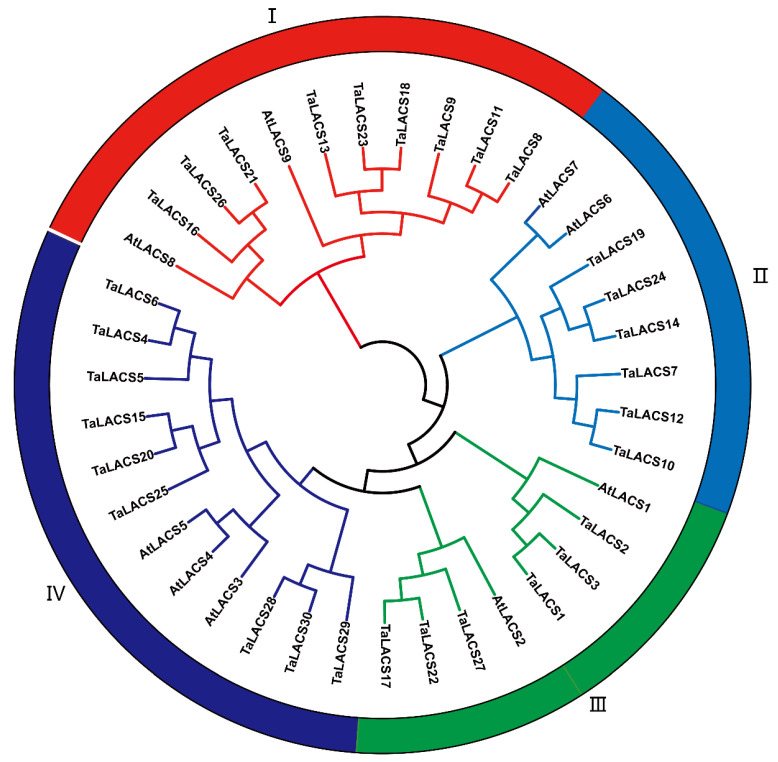
Phylogenetic tree of the wheat and Arabidopsis LACS family. An un-rooted neighbor joining (NJ) tree for LACS proteins was constructed, based on the amino acid sequence alignments of wheat and Arabidopsis LACS. All the proteins are clustered into four clades shown in different circles with different colors.

To detect the evolutionary relationships of LACS proteins among wheat and Arabidopsis, an un-rooted neighbor joining (NJ) tree was constructed using the full-length amino acid sequences of nine Arabidopsis and 30 wheat LACS proteins (Figure 1). The phylogenetic analysis revealed that the LACS could be naturally grouped into four major clades, named Clade I to IV. These clades contained 9, 6, 6, and 9 wheat LACS proteins, respectively. There are 2, 2, 2, and 3 *AtLACS* in clade I to IV, respectively. All of the wheat LACS clustered together with three highly homologous genes.

### 2.3. Motif Composition and Gene Structure of The TaLACS Genes

**Figure 2:**
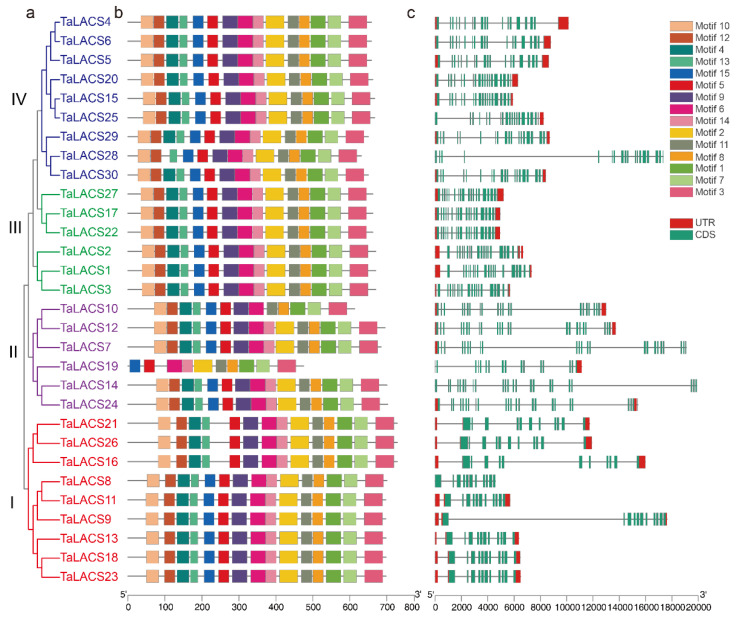
Phylogenetic relationship, conserved motif, and gene structure analysis of *TaLACS* genes. (**a**) Phylogenetic tree of wheat LACS proteins. (**b**) Conserved motifs of wheat LACS proteins. (**c**). Exon-intron structures of *TaLACS* genes. Fifteen conserved motifs are shown in different colored boxes, as indicated on the right of the figure. Green boxes in (**c**) represent exons, black lines represent introns, and the upstream/downstream regions of *TaLACS* genes are represented by red boxes.

To further understand the evolutionary characteristics of the LACS gene family in wheat, the protein motif and gene structure were analyzed in this study. A phylogenetic tree was constructed, using the full amino acid of wheat LACS proteins. The conserved motifs of 30 wheat LACS proteins were analyzed, using the MEME Suite tool. This analysis generated 15 putative conserved motifs for all wheat LACS proteins. As shown in Figure 2, motifs of all the wheat LACS proteins were classed into four clades. There are 15 conserved motifs for proteins in clade III (green) and IV (blue). All six proteins in clade II (purple) have 15 motifs except *TaLACS9* and *TaLACS10*. Motif 12 was not found in *TaLACS10*. Motifs 4, 9, 10, 12, and 13 were not found in *TaLACS9*. Most proteins in clade I (red) have 15 motifs except *TaLACS16*, *TaLACS21*, and *TaLACS26*. Motif 15 was not found in these proteins. The exon/intron structures of *LACS* genes were further analyzed and presented (Figure 2b). The exon/intron structures in the *TaLACSs* varied among different clusters, but were relatively conserved within the same cluster. All of the *TaLACSs* in clade I contain 10 exons. *TaLACSs* in clade II contain 16–23 exons, with 16 exons in *TaLACS19*, and 21 exons in *TaLACS10*. There are 23 exons in *TaLACS7*, *TaLACS10*, *TaLACS14*, and *TaLACS24* proteins. All the proteins in clade III contain 19 exons. All of the proteins in clade IV contain 19 exons except *TaLACS28*, which includes 18 exons.

### 2.4. Chromosomal Location and Gene Synteny Analysis

**Figure 3:**
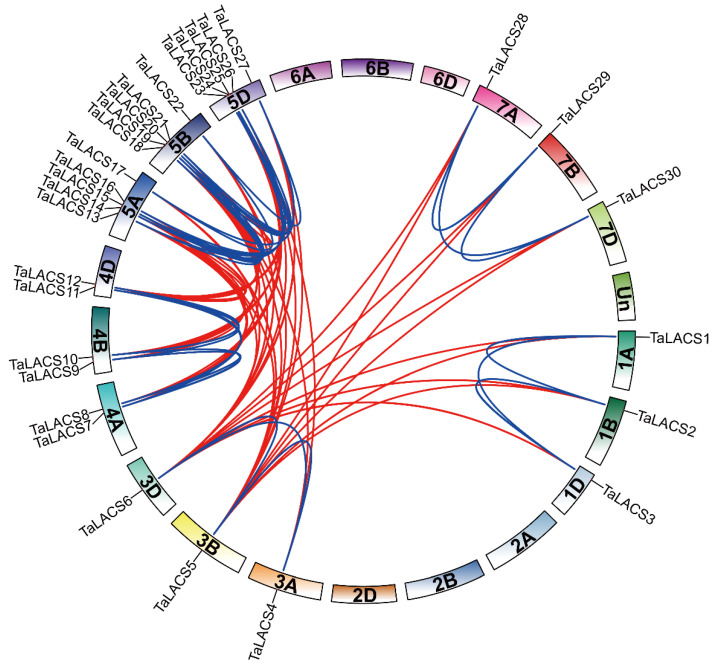
Synteny relationships of LACS genes in wheat. The outer circle segment represents the wheat chromosomes including “unchromosomes”. Un denotes genes with unknown physical positions. Link lines in the circle represents segmental duplication pairs between *TaLACSs*. The blue and red lines represent the segmental duplication pairs between the homologous and paralogous *TaLACSs*.

In line with the available wheat genome annotation information, a total of 30 LACS genes were mapped onto 15/21 chromosomes. All the *TaLACSs* were present in triads. LACS genes were evenly distributed on the wheat chromosomes. There are ten *TaLACSs* located in the A, B, and D sub-genome, respectively. The number of *TaLACSs* on each chromosome was different. As shown in Figure 3, there are one, one, two, five, and one *TaLACS* genes located on linkage one, three, four, five, and seven for the A, B, and D sub-genome, respectively. More than one *TaLACSs* was found on chromosome 4A, 4B, and 4D, and 5A, 5B, and 5D (Figure 3). Tandem duplications were characterized as multiple members of one family occurring within the same intergenic region or in neighboring intergenic regions [22]. According to previous research, a tandem duplication event is defined when there are two or more genes inside 200 kb [23]. As a result, two genes in linkage four and five genes in linkage five were not identified as tandem repeats. These results indicate that tandem duplications might not contribute to the expansion of this gene family.

### 2.5. Cis-Acting Elements in the Promoters and GO Annotations of TaLACSs

**Figure 4:**
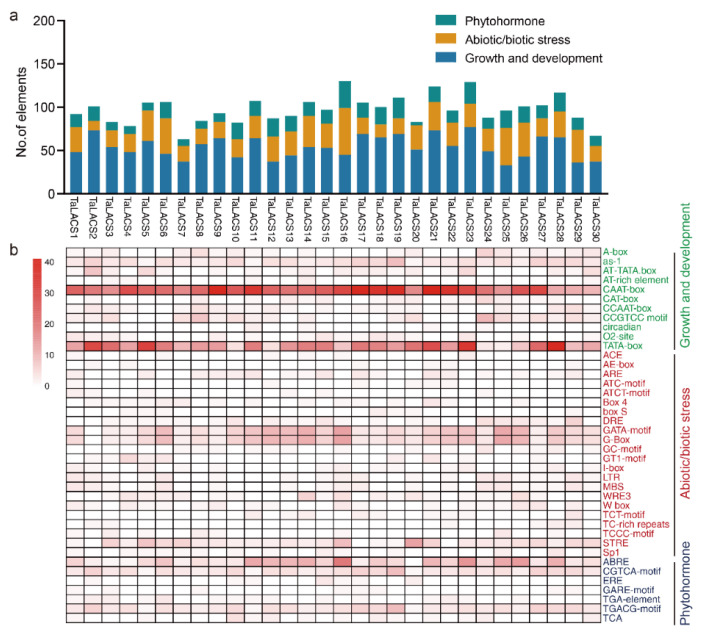
*Cis*-acting elements in the promoters of wheat *TaLACS* genes. (**a**) Number of elements assigned to biotic/abiotic stress, growth and development, and phytohormone responses for each *TaLACS*. (**b**) *Cis*-acting elements in the promoters of *TaLACS* genes in wheat. The different shades of red represent the number of *cis*-acting elements.

*Cis*-acting element prediction in the promoters and Gene Ontology (GO) annotation will be of much help in the understanding of the *TaLACS* functions in wheat. Transcription factors (TFs) represent an important group of regulators involved in the regulation of gene expression at the transcriptional level. *Cis*-acting elements in the promoter are crucial regions of the binding site of the transcription factors for initiating transcription and gene expression. To gain an insight into the potential regulatory mechanism of TFs in the expression of *LACS*, the 2000 bp upstream promoter regions of all *TaLACSs* were used to predict the *cis*-acting regulatory elements via PlantCARE. All the *cis*-acting elements in the promoters of *TaLACSs* were classified into three categories, including phytohormone, abiotic/biotic stress, and growth and development (Figure 4a). The average number of *cis*-acting elements related to growth and development were the highest (54), followed by abiotic/biotic stress (27), and phytohormone (16). The top three genes with most *cis*-acting elements were *TaLACS21*, *TaLACS19*, and *TaLACS23* in growth and development, *TaLACS6*, *TaLACS25*, and *TaLACS16* in abiotic/biotic stress, and *TaLACS16*, *TaLACS19*, and *TaLACS23* in phytohormone (Figure 3a). *Cis*-acting elements involved in growth and development, including CAAT-box, TATA-box, as-1, CCGTCC motif, A-box, and CCAAT-box were found in the promoter sequences of *TaLACSs*. As shown in Figure 4b, *cis*-acting elements including CAAT-box and TATA-box were found in the promoters of all the *TaLACSs*. CCAAT-box and as-1 were identified in the promoters of 22 and 20 *TaLACSs*, respectively. *Cis*-acting elements involved in abiotic/biotic stress, including GATA-motif, G-Box, STRE, DRE, WRE3, ARE, LTR, and MBS were also found in the promoter sequences of *TaLACSs*. In total, 29 *TaLACSs* contain GATA-motif and G-Box. 27 and 21 *TaLACSs* contain STRE and DRE, respectively. *Cis*-acting elements involved in the phytohormone responses were also found in the promoters of *TaLACSs*. MeJA-responsive elements CGTCA-motif and TGACG-motif were found in all the *TaLACSs’* promoter sequences. Abscisic acid responsive element, ABRE, was found in the promoter sequences of 29 wheat LACS genes. The TGA-element involved in auxin responsiveness was found in the promoter sequences of 15 genes (Figure 4b). MYB and MYC elements were found in all the promoter sequences of *TaLACSs*, which may suggest that the expression of *TaLACSs* may be regulated by MYB/MYC to participate in corresponding biological processes (Supplementary Table S2).

In this study, GO annotation was carried out for all the *TaLACSs*. There are 29, 27, and 7 genes assigned to the biological process, molecular function, and cellular component, respectively. *TaLACSs* were annotated in biological processes including the cutin biosynthetic process, the fatty acid metabolic process, the long-chain fatty acid metabolic process, the phenylpropanoid metabolic process, the defense response to fungus, and lateral root formation. The majority of *TaLACSs* (21/30) were assigned to the fatty acid metabolic process. *TaLACSs* were predicted to function in cellular components such as the endoplasmic reticulum, plasmodesma, membrane, and integral components of the membrane. *TaLACSs* were predicted to have 4-coumarate-CoA ligase activity, trans-cinnamate-CoA ligase activity, long- or very-long-chain fatty acid-CoA ligase activity, ATP-binding, catalytic activity, and nucleotide-binding activity (Supplementary Table S3).

### 2.6. The Expression of TaLACS Genes in Wheat

**Figure 5:**
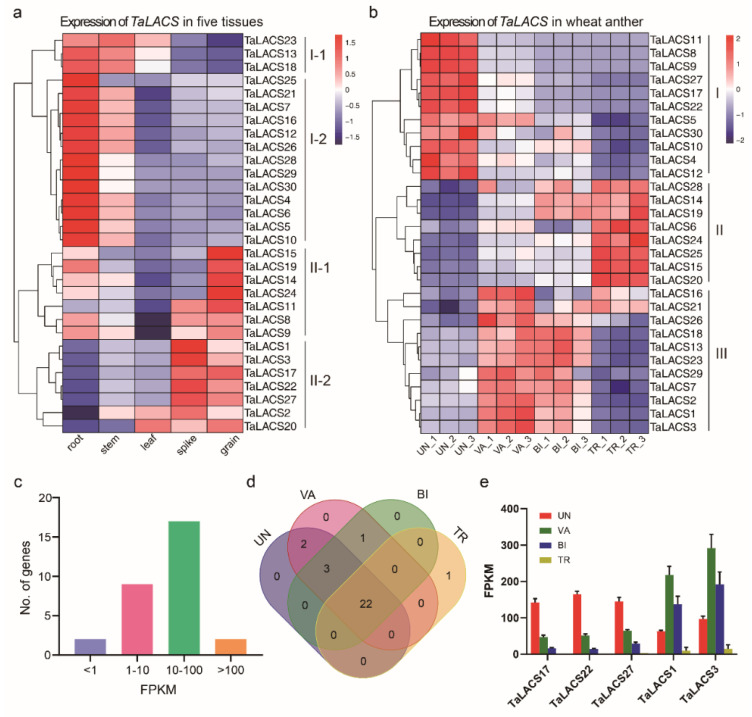
Expression of *TaLACSs* in wheat. (**a**) Expression heatmap of *TaLACSs* in five wheat tissues. (**b**) Expression heatmap of *TaLACSs* genes in wheat anthers. (**c**) The expression range of *TaLACSs* in wheat anther at four stages. (**d**) Venn diagram of *TaLACSs* with expression level higher than 1 FPKM in wheat anthers. (**e**) The expression of the top five expressed *TaLACSs* in wheat anthers. UN, uninucleate stage; VA, vacuolated stage; BI, binucleate stage; TR, trinucleate stage.

To gain a general understanding of the *TaLACS* expression in different tissues, the expression data from five wheat tissues (root, stem, leaf, spike, grain) of Chinese Spring were used in this study. In total, 29 genes were found to express in at least one tissue (FPKM > 1). The number of the expressed *TaLACSs* ranged from 22 (leaf) to 28 (stem) in different tissues. There was only one tissue-specific gene among all the *TaLACS* genes. *TaLACS25* expressed only in root tissue. *TaLACS28*, *TaLACS29*, and *TaLACS30* expressed in both root and stem tissues. *TaLACS11* expressed in grain, spike, and stem tissues. *TaLACS17*, *TaLACS27*, and *TaLACS22* expressed in grain, leaf, spike, and stem tissues. *TaLACS7* and *TaLACS9* expressed in grain, root, spike, and stem tissues. The remaining 19 genes expressed in all five tissues. Based on the expression patterns from five tissues in Chinese Spring, *TaLACS* genes can be classified into two clusters (Figure 5a). Genes in cluster I expressed highly in root, stem, and leaf tissues. Genes in cluster II showed high expression levels in the spike and grain tissues. Genes in clusters I and II can be further classified into two subclusters. Genes in subcluster I-1 are highly expressed in the root, stem, and leaf tissues, while genes in subcluster I-2 were abundantly expressed in the root and stem tissues. Genes in subcluster II-1 were most abundant in the grain tissue. Genes in subcluster II-2 were more abundant in the spike and grain tissues compared with the other three tissues. All of these results suggest that *TaLACSs* might participate in many aspects of biological functions in different tissues, as they express in all five plant tissues.

To further explore the potential roles of *TaLACS* in anther and pollen development, the expression of all the *TaLACSs* was examined in the transcriptome sequencing data of anthers from the early uninucleate, vacuolated, binucleate, and trinucleate pollen stages. Heatmaps of *TaLAC* genes were generated using the expressed genes (average FPKM > 1). As shown in Figure 5b, the expression of expressed *TaLACS* genes can be clustered into three clusters. Genes in cluster I express highly at the uninucleate stage. Genes in cluster II are mainly expressed at the middle two stages, with higher expression at the vacuolated stage and moderate expression at the binucleate stage. Genes in class III are highly expressed at the last stage. These results indicate that *TaLACS* may be important for four respective development stages in anther or pollen development. Among 30 *TaLACSs*, the average expression of 2 *TaLACS* genes was lower than 1 FPKM; the expression of nine genes ranged from one to ten FPKM; the expression level of 17 genes ranged from 10 to 100 FPKM; the expression of two genes was higher than 100 FPKM (Figure 5c). The number of genes expressed were 27, 28, 26, and 23 for the early uninucleate, vacuolated, binucleate, and mature pollen stages, respectively. A total of 22 genes were found expressed in four stages (Figure 5d). Only one gene, *TaLACS28*, was mature pollen-stage specific. *TaLACS29* expressed at both the vacuolated and binucleate stages. *TaLACS11* and *TaLACS9* expressed at both the uninucleate and vacuolated stages. *TaLACS17*, *TaLACS8*, and *TaLACS7* expressed at three early stages. Five genes were highly expressed in wheat anthers (FPKM > 100), with three genes (*TaLACS17*, *TaLACS22*, and *TaLACS27*) at the early uninucleate stage, and two genes (*TaLACS1* and *TaLACS3*) at the middle two stages (Figure 5e). All these results indicate that *TaLACS* genes may participate in late anther development, during which the microspores develop into mature pollen grains.

### 2.7. Correlation between TaLACSs and Transcription Factors in Wheat Anther

**Figure 6:**
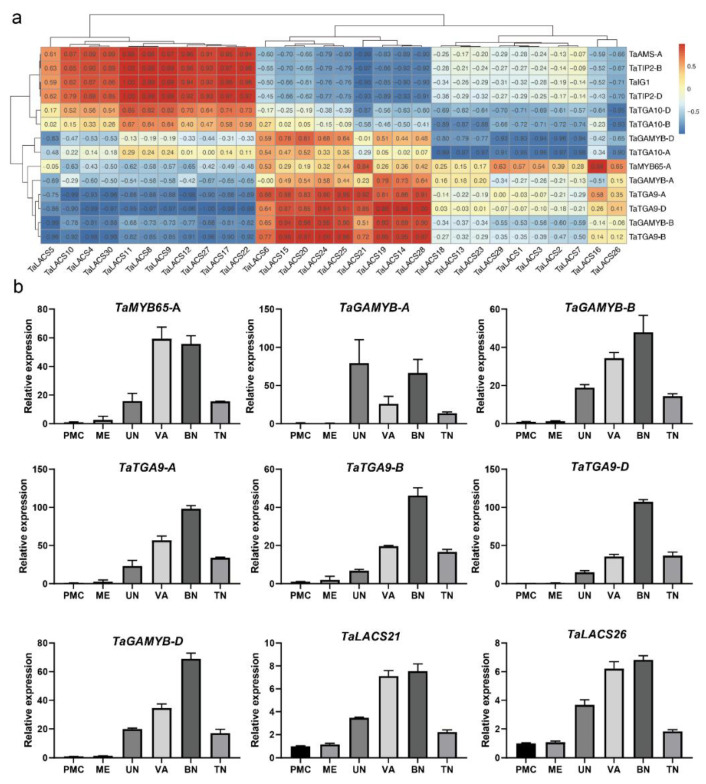
Expression correlation analysis among *TaLACS* genes and transcription factors in wheat anther. (**a**) Expression correlation analysis among *TaLACS* genes and transcription factors. Red indicates positive correlation; blue indicates negative correlation. Pearson correlation coefficients between genes were shown in the boxes of heatmap. (**b**) Expression analysis of selected transcription factors and *TaLACS* in wheat anthers.

To further study the role of *TaLACS* in wheat anther development, transcription factors reported to be involved in anther development were identified in the wheat anther transcriptome sequencing data. *TaTGA9*-*B*, *TaTGA9*-*A*, *TaTGA9*-*D*, *TaTGA10*-*D*, *TaTGA10*-*B*, and *TaTGA10*-*A* are orthologs of the *AtTGA9* and *AtTGA10*, which encode a basic leucine-zipper (bZIP) transcription factor. It has been reported that *AtTGA9* and *AtTGA10* are redundantly required for anther development [24]. *TaIG1*-*B* is an ortholog of maize *IG1*, which encodes a LOB domain (LBD) transcription factor [25]. *TaAMS*-*A* is an ortholog of *ABORTED MICROSPORE* (*AMS*) in Arabidopsis [26]. *TaTIP2*-*B* and *TaTIP2*-*D* are orthologs of *TIP2* in rice [27]. *TaAMS*-*A*, *TaTIP2*-*B*, and *TaTIP2*-*D* all encode the basic helix-loop-helix (bHLH) transcription factors. *TaGAMYB*-*D*, *TaGAMYB*-*A*, and *TaGAMYB*-*B* are orthologs of *HvGAMYB* in barley [28]. *TaMYB65*-*A* encodes a protein orthologous to *MYB DOMAIN PROTEIN 65* (*MYB65*) in Arabidopsis [29]. The correlation between those 14 transcription factors and *TaLACSs* in the wheat anthers was analyzed (only |Pearson correlation coefficient| higher than 0.8 was considered here) (Figure 6a). The correlations between *TaLACSs* and transcription factors were clustered into three sections. In the first cluster, *TaLACSs* were positively correlated with *TaIG1*-*B*, *TaTIP2*-*B*, *TaTIP2*-*D*, and *TaAMS*-*A*, and negatively correlated with *TaTGA9*-*B*, *TaTGA9*-*A*, *TaTGA9*-*D*, and *TaGAMYB-B*. The correlations in the second cluster were the opposite. *TaLACSs* in the second cluster were positively correlated with *TaTGA9*-*B*, *TaTGA9*-*A*, *TaTGA9*-*D*, and *TaGAMYB-B*, and negatively correlated with *TaIG1*-*B*, *TaTIP2*-*B*, *TaTIP2*-*D*, and *TaAMS*-*A*. *TaLACS* in the third cluster were negatively correlated with *TaTGA10*-*A*, *TaTGA10*-*B*, *TaTGA10*-*D*, and *TaGAMYB-D*. *TaMYB65*-*A* was positively correlated with only two genes (*TaLACS16* and *TaLACS21*).

To further investigate the possible roles of *TaLACS* in anther development, quantitative RT-PCR (qRT-PCR) was used to measure the expression patterns of selected *TaLACS* and potential correlated transcription factors in the wheat anthers (Figure 6b). The expression of transcription factors including three *TaTGA9*, three *TaGAMYB*, and *TaMYB65-A* were examined in this study. The expression of transcription factors including *TaMYB65-A*, *TaGAMYB*-*D*, *TaGAMYB-B*, and three *TaTGA9* increased gradually with the extension of time and decreased at the trinucleate stage. *TaMYB65-A* expressed at the highest level at the vacuolated and binucleate stages. *TaGAMYB-A* expressed at the highest level at the uninucleate and binucleate stages. The expression of *TaGAMYB*-*D*, *TaGAMYB-B*, and three *TaTGA9* coding genes started to increase from the uninucleate stage, with the highest expression level at the binucleated stage. *TaLACS21* and *TaLACS26* up-regulated from the uninucleate stage to the binucleated stage. The similar expression pattern between *TaLACSs* and TFs, including three *TaTGA9* and *TaGAMYB-B,* suggested their positive correlation roles in late anther and pollen development.

### 2.8. Expression of TaLACS in the Temperature-Sensitive Genic Male-Sterile Line

**Figure 7:**
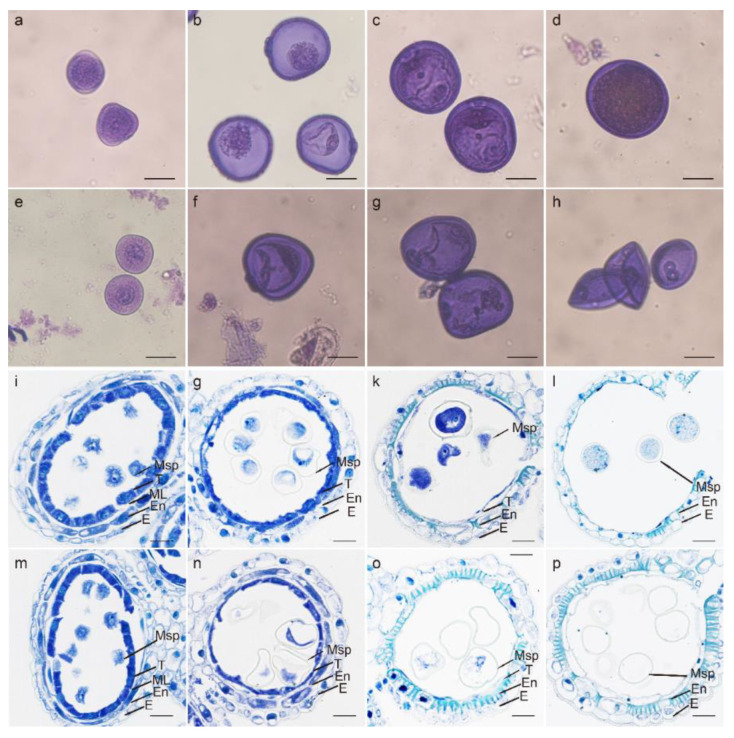
The microspores and anthers at different developmental stages. Microspores at early uninucleate stage (**a**,**e**), vacuolated stage (**b**,**f**), binucleate stage (**c**,**g**), and mature pollen stage (**d**,**h**). Wheat anther at early uninucleate stage (**i**,**m**), vacuolated stage (**g**,**n**), binucleate stage (**k**,**o**), and mature pollen stage (**i**,**p**). (**a**–**e**,**i**–**l**), pollens and anthers of control-treated BS366; (**e**–**h**,**m**–**p**), pollens and anthers of cold-treated BS366; E, epidermis; En, endothecium; ML, middle layer; Msp, microspores; T, tapetum; bars in (**a**–**h**) 20 μm; bars in (**i**–**p**) 50 μm.

Wheat temperature-sensitive genic male-sterile (TGMS) line BS366 (Beijing Sterility 366), is normal at 20 °C (control) but produces sterile pollen at 12 °C (cold) with 12 h of daylight. At the early uninucleate stage, free microspores are released from the tetrads. Microspores are spherical with thin exines (Figure 7a,e). Tapetal cells reabsorb their vacuoles, and the cytoplasm becomes condensed (Figure 7i,m). No differences were observed between the cold- and control-treated BS366. At the vacuolated stage, thicker exine are formed on the outer surface of the microspores. The microspore vacuolates with an increase of volume, resulting in a round-shaped microspore (Figure 7b). Tapetal cells become more degenerated, and the middle layer becomes invisible (Figure 7g). However, the pollen grains of cold-treated BS366 were irregularly spherical (Figure 7f,n). At the binucleate stage, the vacuolated microspore undergoes the first mitotic division with asymmetric cell division, generating a much smaller generative cell and a larger vegetative cell. As the starch accumulates inside the microspore, the vacuole diminishes gradually (Figure 7c). The tapetum cells almost completely degenerated (Figure 7k). In the cold-treated pollens, no starch accumulated in the pollen grain and the endothecium became thicker compared with the control-treated BS366 (Figure 7g,o). At the mature stage, the fertile pollen grains are full of starch (Figure 7d). The epidermis and the endothecium degenerate further, and the tapetum completely disappears (Figure 7i). Anther dehiscence occurs and mature pollen grains are released (Figure 7i). Pollens in the cold-treated BS366 were vacuolated and shrank (Figure 7h). The endothecium became abnormally expanded and thicker (Figure 7p).

**Figure 8:**
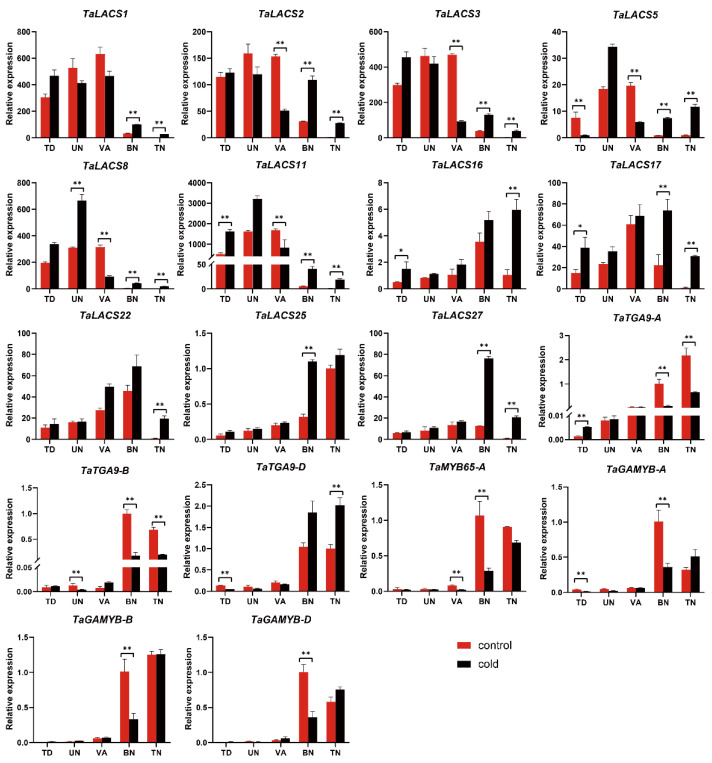
Expression of LACS genes and transcription factors in BS366 at different developmental stages between cold and control conditions. Asterisks indicate significant differences between cold and control conditions (Student’s *t*-test, * *p*-value < 0.05, ** *p*-value < 0.01). TD, tetrad stage; UN, uninucleate stage; VA, vacuolated stage; BN, binucleate stage; TN, trinucleate stage.

Pollen development after meiosis involves pollen exine development, starch accumulation, and nuclear mitosis. It has been reported that lipid metabolism plays an important role in pollen exine formation. To explore the roles of *TaLACS* in BS366 during pollen and late anther development, the expression of 11 *TaLACSs* and *TaTGA9*-*D* were studied in BS366 anthers under the cold and control conditions. As the pollen exine formation takes place before the early uninucleate stage, the expression of those genes was checked at the tetrad stage. As shown in Figure 8, *TaLACS1*, *TaLACS2*, and *TaLACS3* are three orthologs of *AtLACS1*. The expression of the three genes started to accumulate from the early uninucleate stage, and decreased at the binucleate stage. *TaLACS2* and *TaLACS3* were significantly repressed at the vacuolated stage, but all three *TaLACSs* were induced at the binucleate and trinucleate stages. *TaLACS5* was homologous to *AtLACS5.* It was differentially expressed at all five stages. The expression of *AtLACS5* was repressed under cold conditions at both the tetrad and vacuolated stages, but induced at the other three stages. *TaLACS8* and *TaLACS11* were two wheat homologues and clustered together with *AtLACS9*. Both genes were induced in the cold-treated BS366 at all stages except the vacuolated stage. *TaLACS16* was clustered together with *AtLACS8*. It was differentially regulated at both the tetrad and trinucleate stages. Although *TaLACS17*, *TaLACS22*, and *TaLACS27* were three wheat homologues, the expression patterns were different for the three genes. The expression of the three genes increased from the tetrad stage and peaked at the binucleate stage. All three genes were induced at the trinucleate stage. *TaLACS17* and *TaLACS27* were both induced by the cold treatment. Only *TaLACS17* was significantly induced at the tetrad stage. The expression of *TaLACS25* increased over time in the cold-and control-treated BS366, but the expression of *TaLACS25* was higher under the cold condition at the binucleate stage. The expression of transcription factors in Figure 6 were also examined in the cold- and control-treated BS366. The expression of *TaTGA9*-*A* and *TaTGA9*-*B* was similar. The expression of both genes was repressed at two later stages in the cold-treated BS366. However, the expression of *TaTGA9*-*D* peaked at the last two stages and was induced by the cold treatment. The expression of *TaTGA9-D* was repressed at the tetrad stage, but increased at the trinucleate stage. The expression of *TaMYB65*-*A* was significantly repressed at the vacuolated and binucleate stages. *TaGAMYB-A* was repressed at the tetrad stage. In addition, all three *TaGAMYB* transcription factors were repressed at the binucleate stage in BS366 under cold conditions. All the differential regulation of the LACS genes and transcription factors in BS366 between the cold and control conditions suggested their potential roles in the male sterility of BS366.

## 3. Discussion

In flowering plants, the life cycle alternates between diploid sporophyte and haploid gametophyte generations. Male gametophytes in wheat develop from the initiation and generation of the male reproductive structure stamen, which consists of three anthers and filaments supporting each anther. Anthers are the male reproductive organs that generate pollen grains. The reproductive stage is directly related to seed production and life continuation. Understanding the molecular mechanism of anther and pollen development is crucial for future hybrid breeding [30]. During anther development, tapetum formation and apoptosis, meiosis, callose generation and degradation, and pollen wall formation, are closely related to pollen development [31]. Fatty acids and their derivatives are essential components of anther cuticle and pollen wall development. LACS responsible for the conversion of free fatty acids into acyl-CoAs, are involved in multiple pathways of lipid metabolism, such as fatty acid transport, lipid synthesis, and fatty acid β-oxidation [5,6].

To date, functional research on LACS genes has been conducted in many species, proving the critical roles of LACSs in fatty acid metabolism [6,32,33,34]; however, few related works have been applied to wheat. In this study, a genome-wide identification and expression analysis in the anther were carried out in common wheat. Finally, a total of 30 LACS genes were identified in the wheat genome. The identification of wheat LACS genes and their homologous relationships between *AtLACS* will be helpful in the understanding of their corresponding roles in wheat. Arabidopsis contains nine LACS genes, seven of which have been well characterized [1]. In this study, *TaLACS1*, *TaLACS2,* and *TaLACS3* clustered together with *AtLACS1*. *TaLACS17*, and *TaLACS22*, and *TaLACS27* clustered with *AtLACS2*. It has been reported that *AtLACS1* was involved in wax synthesis, and the mutation of *AtLACS1* caused a strong deficient glossy phenotype [7]. *AtLACS2* is mainly involved in cutin biosynthesis. The mutation of *AtLACS2* caused strong cutin deficiency, but had little effect on wax biosynthesis under normal growth condition [35]; however, it has been reported that *AtLACS2* is also involved in wax synthesis under stress conditions [36]. However, recent studies reported that *AtLACS2* appears to function overlapping with *AtLACS1* in wax and cutin production [9,14]. The anther cuticle is an extracellular lipidic layer that covers the anther surface and protects anthers from external abiotic stresses, water loss from the inner tissues, and attack by pathogens [37,38]. It is composed of a cutin polymer matrix and waxes [37]. In plants, cuticular wax, cutin, and suberin are commonly defined as surface-covering lipids [37]. Cutin is a crosslinked, amorphous, and viscoelastic polymer formed almost exclusively by the interesterification of C16- and C18-polyhydroxy fatty acids [39]. Although the roles of *AtLACS1* and *AtLACS2* in anther and pollen development have not been reported, we may speculate the role of wheat orthologs of both genes in anther and pollen development. In this study, the expression of *TaLACS1*, *TaLACS2,* and *TaLACS3* was detected at the three early stages. However, the expression of *TaLACS1* was significantly higher at the two later stages. *TaLACS2* and *TaLACS3* were repressed at the vacuolated stage and induced at the binucleate and trinucleate stages under the cold condition. These results suggested that *TaLACS2* and *TaLACS3* may participate in wheat anther development. The expression of *TaLACS17* was significantly higher than that of the cold-treated BS366 at the tetrad, binucleate, and trinucleate stages. *TaLACS27* was significantly induced at the latter two stages, while *TaLACS22* was only induced at the trinucleate stage in the cold-treated BS366, compared with the control-treated BS366. As the expression of the three LACS genes increased from the tetrad stage, we may conclude that the three genes may participate in late anther development and pollen exine formation. The differential expression of *TaLACS17*, *TaLACS22*, and *TaLACS27* at the late stages suggest that male sterility in BS366 might have an effect on the fatty acid metabolism in the later anther development.

Three homoeologous genes, *TaLACS16*, *TaLACS21*, and *TaLACS26* were clustered together with *AtLACS8*. *TaLACS8*, *TaLACS9*, *TaLACS11*, *TaLACS13*, *TaLACS18*, and *TaLACS23* were clustered together with *AtLACS9*. *AtLACS8* was reported to be functionally overlapped with *AtLACS4* and *AtLACS9* in the lipid trafficking between the ER and plastid for glycerolipid synthesis [1]. In this study, the expression of *TaLACS11* and *TaLACS16* was significantly higher at the tetrad stage in BS366 under the cold condition than that under the control condition. *TaLACS8* were significantly induced at the uninucleate stage in the cold-treated BS366. Although *AtLACS9* functions redundantly with either *AtLACS1* or *AtLACS4* in seed TAG biosynthesis, the differential expression of homologous wheat LACS in this study might suggest their roles in wheat male sterility [12,40]. Three LACS in Arabidopsis clustered together in the phylogenetic analysis, including *AtLACS3*, *AtLACS4*, and *AtLACS5*. The function of *AtLACS5* is still unclear. It has been suggested that *AtLACS5* is exclusively expressed in anthers [16]. *GhACS1*, an ortholog of the Arabidopsis *AtLACS4* or *AtLACS5*, is predominantly accumulated in the anther and plays an essential role in microsporogenesis in the anther development of cotton [19]. *TaLACS5* and *TaLACS25*, two orthologs of the *AtLACS4* or *AtLACS5*, were differentially expressed in the BS366 anther under the cold and control conditions. The expression of *TaLACS5* was repressed at the tetrad and vacuolated stages but induced at two latter stages in BS366 under cold conditions. *TaLACS25* were induced at the binucleate stage in the cold-treated BS366. In addition to the plant cuticle, very-long-chain lipids were also detected in the extracellular pollen coat (tryphine), where they play crucial roles in pollen-stigma communication [9]. Tryphine is mainly composed of complex lipids, wax esters, flavonoids, proteins, pigments, aromatic substances, and other unknown compounds [38,41]. In the mature pollen grain, the tryphine fills the cavities of the pollen exine. Most lipids detected in the tryphine are derivatives of very-long-chain fatty acids (VLCFAs) [42]. It has been reported that *LACS1* and *LACS4* are required for proper pollen coat formation in Arabidopsis [9]. Thus, the differential expression of *TaLACS5* and *TaLACS25* may be correlated with pollen coat formation.

Male reproductive development in higher plants can be regulated by Gibberellins (GA). The transcriptional factor *GAMYB* is a crucial component of GA signaling in anther development. Transgenic barley over-expressing the *HvGAMYB* gene failed to dehisce and were male-sterile compared with non-transgenic controls [28]. It has been reported recently that the silencing of *TaGAMYB* in wheat displayed fertility decline and defects in tapetum, pollen and exine formation. In addition, either the hot temperature or GA3 treatment in YanZhan 4110S caused the down-regulation of *TaGAMYB* at the binucleate stage and trinucleate stage, as well as fertility decrease [43]. In this study, three orthologs of *HvGAMYB* were identified. The expression level of *TaGAMYB*-*A* was highest at the uninucleate and binucleate stages. The expression of *TaGAMYB*-*B* and *TaGAMYB*-*D* started to increase from the uninucleate stage and continued until the binucleate stage (Figure 6). All these results indicated that *TaGAMYB*-*B* and *TaGAMYB*-*D* might play critical roles in anther development from the uninucleate stage. All three *TaGAMYB* transcription factors were significantly repressed at the binucleate stage, which suggested their potential roles in the male sterility of BS366. The expression of *TaGAMYB*-*B* was negatively correlated with the expression of *TaLACS4* and *TaLACS5*. The expression of *TaLACS5* decreased from the binucleate stage. Differential expression analysis of the control- and cold-treated BS366 revealed that the expression of *TaLACS5* was repressed under the cold condition at the tetrad and vacuolated stages. Thus, we may conclude that *TaLACS5* might mainly function in anther development until the vacuolated stage. The repression of *TaLACS5* at the vacuolated stage suggested the defect of BS366 under the cold condition impaired the lipid metabolism which involved the *TaLACS5*. In the *cis*-activating element analysis, MYB and MYC elements were found in all the promoter sequences of *TaLACSs*. Thus, we may conclude that wheat LACS genes including *TaLACS5* may be regulated by the *GAMYB* transcription factors to participate in anther development.

## 4. Materials and Methods

### 4.1. Genome-Wide Identification of LACS Family Genes in Wheat

Nine LACS proteins in Arabidopsis were used in this study. Sequences of nine proteins were downloaded from the Arabidopsis genome database (https://www.Arabidopsis.org/ (accessed on 1 April 2022)) [6]. The BLASTP method was used to identify the candidate LACS proteins in wheat with a cutoff e-value of 1 × 10^−20^ and a score higher than 100 [44]. The HMM profile of the AMP-binding proteins (PF00501) was downloaded from the Protein family database (Pfam, https://pfam.xfam.org/ (accessed on 7 April 2022)). The software HMMER was used to search for the AMP-binding proteins encoding genes against the genome of wheat using the HMM file of AMP-binding with a cutoff of E < 1 × 10^−20^. The conserved domain of the generated AMP-binding proteins in wheat were filtered using the Pfam, SMART (http://smart.embl.de/ (accessed on 8 April 2022)), and CDD (https://www.ncbi.nlm.nih.gov/cdd/ (accessed on 10 April 2022)) in NCBI. All retained proteins were blasted against the Arabidopsis genome to eliminate proteins with other functions. Finally, 30 genes were common to both methods. After excluding splice variants, the longest transcripts of 30 *TaLACSs* were retrieved from wheat. All identified wheat genes were renamed according to their genomic locations. Finally, the acquired sequences were submitted to ExPASy (https://web.expasy.org/protparam (accessed on 11 April 2022)) to calculate the physicochemical parameters such as molecular weight (MW) and theoretical isoelectric point (pI).

### 4.2. Multiple Alignment and Phylogenetic Analysis of the Wheat LACS Family Genes

To determine the evolutionary relationships of the wheat LACS, the amino acid sequences of nine Arabidopsis LACS and 30 wheat LACS were subjected to sequence alignment and phylogenetic tree construction using MEGA7 software. The ClustalW function in MEGA7 was used for sequence alignment. An un-rooted neighbor joining (NJ) tree for LACS proteins in two species was constructed using MEGA7 [45]. An un-rooted neighbor joining (NJ) tree for LACS proteins only in wheat was also constructed in this research.

### 4.3. The Gene Structure and Conserved Domains in Wheat LACS

Fifteen conserved motifs with lengths of 6–50 amino acids were obtained for wheat LACS using meme-5.1.0 (https://meme-suite.org/meme/tools/meme (accessed on 7 July 2022)) [46]. Combined with the wheat genome annotation information, the conserved motifs and intron/exon pattern of the gene structure was determined for wheat LACS. The conserved domain and gene structure was analyzed and visualized with TBtools v1.098767 (https://github.com/CJ-Chen/TBtools/releases (accessed on 22 August 2022)) [47].

### 4.4. Chromosomal Location of TaLACS and Gene Duplication Analysis

To map the putative LACS genes onto the wheat chromosomes, the initial chromosomal position of *TaLACSs* and length of the chromosome were identified from the wheat genome (http://ftp.ensemblgenomes.org/pub/plants/release-54/gff3/triticum_aestivum/ (accessed on 7 July 2022)). Full amino acid sequences of *TaLACSs* were subjected to a multi-sequence blast, and the top four targets with an E-value lower than 1 × 10^−10^ were selected for this study. The chromosomal distribution and collinearity between *TaLACSs* genes were visualized by TBtools v1.098767 (https://github.com/CJ-Chen/TBtools/releases (accessed on 22 August 2022)) [47]. Tandem duplication of events was defined as two or more adjacent homologous genes located on one chromosome without any intervening gene [22].

### 4.5. GO Annotation and Cis-Acting Elements Analysis of TaLACSs

The Gene Ontology (GO) annotations for wheat LACS were carried out using the TBtools [47]. The molecular functions, biological processes, and cellular components were annotated for the wheat LACS. Sequences of 2000 bp upstream of *TaLACSs* promoter were obtained [47]. The *cis*-acting elements in these regions were predicted using plantCARE (https://bioinformatics.psb.ugent.be/webtools/plantcare/html/ (accessed on 1 May 2022)) [48].

### 4.6. Expression Analysis of TaLACSs in Wheat Tissues

To study the expression patterns of all the *TaLACSs*, expression data from five wheat tissues (root, stem, leaf, spike, and grain) in Chinese Spring were obtained from WheatOmics 1.0 (http://202.194.139.32/ (accessed on 5 May 2022)). To further study the potential roles of *TaLACSs* involved in anther and pollen development, the transcriptome sequencing data of anthers at early uninucleate, vacuolated, binucleate, and mature pollen stages were used to examine the expression of all the putative *TaLACSs* in this study.

### 4.7. Phenotypic Analysis of BS366

The wheat temperature-sensitive genic male-sterile (TGMS) line Beijing Sterility 366 BS366, maintained at the Institute of Hybrid Wheat, Beijing Academy of Agriculture and Forestry Sciences, was used in this study. BS366 is normal at 20 °C (control) but produces sterile pollen at 12 °C (cold) with 12 h of daylight. Spikelets of BS366 from the uninucleate to trinucleate stages under the cold and control conditions were sampled and fixed in FAA solution (formaldehyde:glacial acetic acid:50% ethanol = 5:5:9). The pollens were dyed with improved carbol fuchsin solution, as previous reported [21]. Photographs of the microspores and pollen were obtained using an Olympus BX-53 microscope (Tokyo, Japan). For the anther phenotype analysis, the preparation of the transverse sections of anthers was carried out as reported [21]. The anther morphology was analyzed with a scanning electron microscope (HITACHI SU8100).

### 4.8. Plant Materials, Total RNA Isolation, and qRT-PCR Analysis

The anther transcriptome data of a normal inbred line CP1860 maintained at the Institute of Hybrid Wheat, Beijing Academy of Agriculture and Forestry Sciences was used in this study. Anthers of the wheat inbred line CP1860 were sampled at the early uninucleate, vacuolated, binucleate, and trinucleate stages, with three biological replicates. All samples were frozen in liquid nitrogen and stored at −80 °C. Total RNA was extracted using TRIzol Reagent (Invitrogen Corp., Carlsbad, CA). The concentration and quality was determined with a Nanodrop spectrophotometer and 1% agarose gel electrophoresis. All samples were sequenced using the MGISEQ-T7 platform. Raw reads were filtered to obtain high-quality reads, by removing low-quality reads containing more than 30% bases with Q < 20. After trimming low-quality bases (Q < 20) from the 5′ and 3′ ends of the remaining reads, the resulting high-quality clean reads in each sample were mapped onto the wheat reference genome using HISAT 2.2.1 release 7 (https://daehwankimlab.github.io/hisat2/ (accessed on 7 August 2020) [49]. Only reads that could be mapped onto only one location in the reference genome (unique hits) were kept for further analysis. Fragments per kilobase of exon model per million mapped reads (FPKM) was used to estimate the transcript expression levels in all samples.

For real-time qRT-PCR, cDNA was synthesized according to the manufacturer’s instructions (PrimeScript™ RT reagent Kit with gDNA Eraser, Takara Bio Inc., Shiga, Japan). The expression of *TaLACSs* was quantified with a CFX96 Touch™ Real-Time PCR Detection System (Bio-Rad Laboratories, Hercules, CA, USA) using SYBR Green II (Takara Bio Inc., Shiga, Japan). The expression levels of mRNAs in samples were normalized using the endogenous wheat actin gene with primer sequences 5′-TACTCCCTCACAACAACCG-3′ and 5′-AGAACCTCCACTGAGAACAA-3′. The relative expression levels were calculated using the 2^−ΔΔCt^ method. Primer sequences were designed using Primer3 input version 4.0 (http://primer3.ut.ee/ (accessed on 20 May 2022)). Primers for expression validation are listed in Supplementary Table S4.

## 5. Conclusions

In conclusion, a total of 30 LACS family genes were identified in the wheat genome. The chromosomal location, phylogenetic classification, gene structure, gene duplication, functional domains, and conserved motifs were investigated. The expression assay of all the candidate *TaLACSs* in five wheat tissues and anthers at different developmental stages were analyzed. The correlation of *TaLACSs* between TFs function in anther development was calculated. The expression of several *TaLACSs* were quantified in the cold- or control-treated BS366, a TGMS line. We may conclude that wheat orthologs of *AtLACS2*, *AtLACS3*, and *AtLACS5* might participate in wheat anther development. Results presented here will be of great value to further characterize the biological roles of LACS in male fertility.

## Data Availability

Transcriptome sequencing data for wheat anthers can be found in the National Genomics Data Center (https://bigd.big.ac.cn/, accessed on 10 September 2022) under accession number CRA008140.

## Supplementary Materials

The following supporting information can be downloaded at: https://www.mdpi.com/article/10.3390/ijms231911942/s1.
